# Incidence of major and clinically relevant non-major bleeding in patients prescribed rivaroxaban for stroke prevention in non-valvular atrial fibrillation in secondary care: Results from the Rivaroxaban Observational Safety Evaluation (ROSE) study

**DOI:** 10.1371/journal.pone.0240489

**Published:** 2020-10-09

**Authors:** Alison Evans, Miranda Davies, Vicki Osborne, Debabrata Roy, Saad Shakir

**Affiliations:** 1 Drug Safety Research Unit, Southampton, United Kingdom; 2 University of Portsmouth, Portsmouth, United Kingdom; Ohio State University, UNITED STATES

## Abstract

**Introduction:**

Although the direct oral anticoagulant rivaroxaban is recommended for stroke prevention in patients with non-valvular atrial fibrillation based on Phase III clinical trials, there is still a need for additional safety data from everyday clinical practice. The ROSE study was initiated to collect further information on the safety and utilisation of rivaroxaban in a broader range of patient groups in routine clinical practice.

**Methods and results:**

The ROSE study was conducted in hospitals in England and Wales. Consenting adults with non-valvular atrial fibrillation newly started on rivaroxaban were eligible and followed up for 12 weeks. Data was derived through secondary use of medical records. The primary outcome was major bleeding within gastrointestinal, urogenital and intracranial sites. A total of 4846 patients were enrolled in the study September 2013 to January 2016, 965 of which were treated with rivaroxaban for non-valvular atrial fibrillation. The median age in the rivaroxaban non-valvular atrial fibrillation cohort was 76 years, 53.6% were male. The median HAS-BLED score was 2 and the median CHA_2_DS_2_-VASc score was 4. The risk of major bleeding within each of the primary sites of gastrointestinal, urogenital and intracranial during the 12 week observation period was low (0.2%; n = 2). The risk of major bleeding in all sites was 1.0% (n = 10) at a rate of 5.5 events per 100 patient years.

**Conclusion:**

In terms of the primary outcome risk of major bleeding within gastrointestinal, urogenital and intracranial sites during the 12 week observation period, the risk estimates in the non-valvular atrial fibrillation rivaroxaban user population were low (<1%), and consistent with risk estimated from clinical trial data and in routine clinical practice.

## Introduction

The prevalence of atrial fibrillation (AF) in the United Kingdom (UK) is increasing across all age groups in both males and females and approximately 3% of the UK population aged ≥ 35 years is diagnosed with AF [1]. The risk of subsequent stroke with AF is increased fivefold and is reduced through use of oral anticoagulation [1, 2]. Direct oral anticoagulants (DOACs) have been introduced over the last decade and are recommended as an alternative to conventional anticoagulant treatment with vitamin K antagonists (VKAs) in international guidelines [2, 3]. The DOAC, rivaroxaban, was shown to be at least as effective as warfarin for the prevention of stroke in non-valvular AF (NVAF) in the pivotal clinical trial and was approved in 2011 for the prevention of stroke and systemic embolism in adult patients with NVAF (with one or more risk factors, such as congestive heart failure [CHF], hypertension, age ≥ 75 years, diabetes mellitus, prior stroke or transient ischaemic attack [TIA]) [4, 5]. Rivaroxaban was also licenced for the treatment of deep vein thrombosis (DVT) and prevention of recurrent DVT and pulmonary embolism (PE) in 2011. The licence was subsequently extended to include the treatment of PE in 2012 [5].

In order to increase knowledge of effectiveness and safety of rivaroxaban in larger groups of patients following the extension of the licence to include NVAF and prevention and treatment of DVT and PE, additional post marketing observational studies were included as part of the European Union (EU) Risk Management Plan (RMP), including two UK-based active pharmacovigilance studies conducted in primary and secondary care [6]. We report on one of these studies, the Rivaroxaban Observational Safety Evaluation (ROSE) study (EU PAS Register Number EUPAS3979), a prospective non-interventional cohort study to evaluate the safety and utilisation in patients prescribed rivaroxaban for the prevention of stroke in patients with NVAF, treatment of DVT and PE, and the prevention of recurrent DVT and PE in a secondary care setting in England and Wales, using the technique of Specialist Cohort Event Monitoring (SCEM) [7]. The SCEM design responds to the requisite for safety surveillance of new medicines initiated in the hospital setting enabling patients to be captured during the early phase of treatment. This will include those who may be more complex in terms of underlying disease, co-morbidities and concomitant medications than the general disease population treated in primary care. SCEM has been developed in parallel with the requirement for pharmaceutical companies to undertake a RMP as part of post-authorisation safety monitoring.

The ROSE study included a contextual cohort of patients prescribed warfarin, in order to compare reasons for choice of anticoagulation type, and to explore differences in both the clinical setting of initiation and baseline risk of bleeding and stroke. Whilst bleed outcomes were also estimated, due to the different eligibility criteria for the rivaroxaban and warfarin cohorts (i.e. based on previous anticoagulant therapy) the study did not allow any direct comparisons between the two cohorts and therefore the warfarin bleed incidence results have not been included here. The present paper focuses on the main clinical endpoint of interest; the incidence of major bleeding within gastrointestinal, urogenital and intracranial sites according to the primary outcome, amongst rivaroxaban users treated for stroke prevention in NVAF, within the first three months. Study results relating to the incidence of major bleeding amongst rivaroxaban users treated for DVT and PE, and the prevention of recurrent DVT and PE, and rivaroxaban utilisation, including use in special populations of patients, will be published separately.

## Methods

### Study design and participants

**Fig 1:**
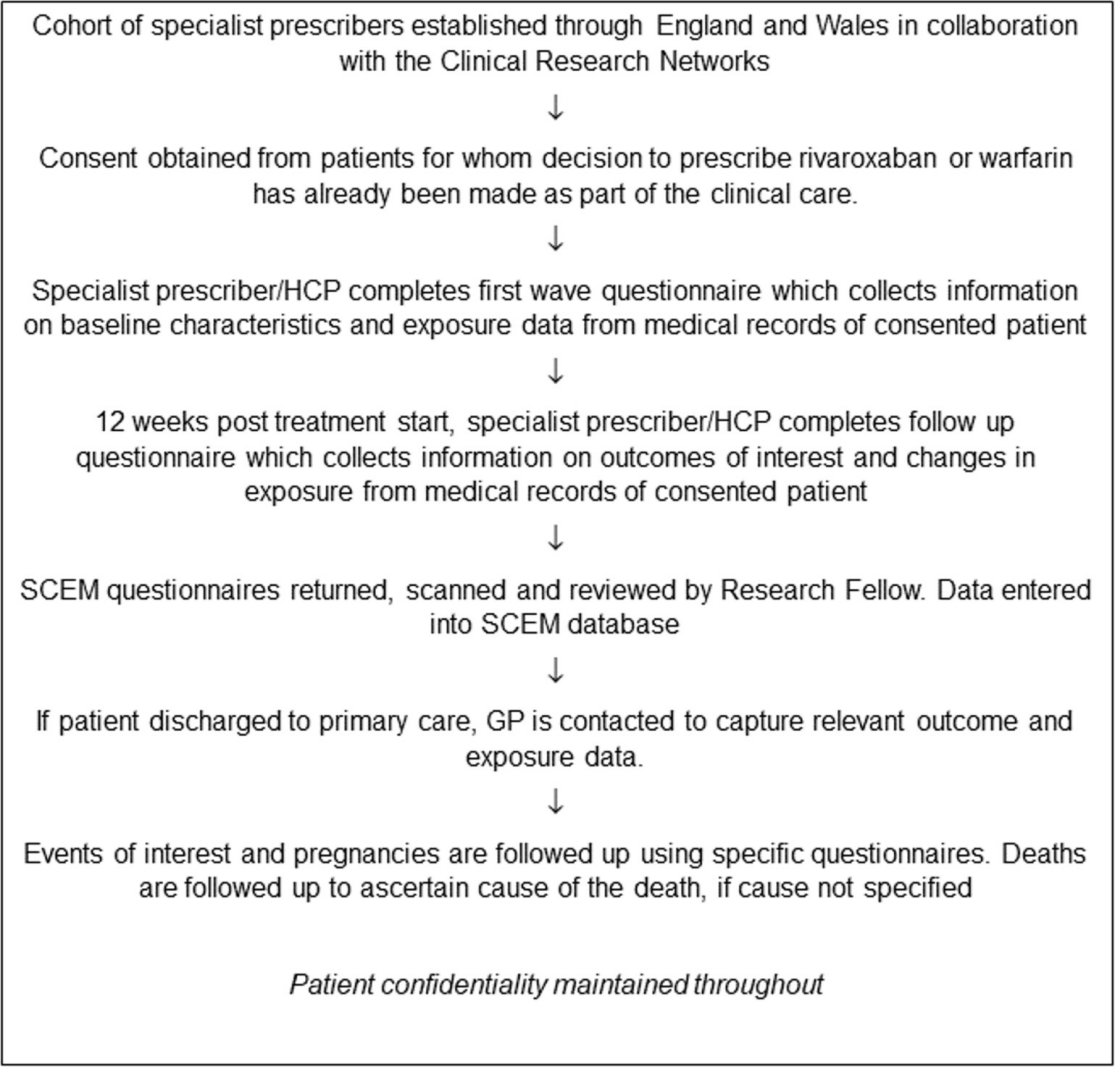
SCEM study process for ROSE. HCP = healthcare professional, GP = general practitioner.

The ROSE study was conducted in the secondary care hospital setting in England and Wales, using the technique of SCEM [7] (Fig 1), as NVAF is most likely to be initially treated within this setting in the UK at the time of conducting the study. Patients were identified through clinical speciality groups during September 2013 to January 2016, supported by UK Clinical Research Networks. All National Health Service (NHS) trusts in England and Wales were invited to participate and study facilitators were available to assist with study implementation.

The study included patients with NVAF treated for prevention of stroke and systemic embolism. Patients were eligible for inclusion if they were at least 18 years old, had provided signed informed consent and were rivaroxaban naïve (no previous use of rivaroxaban). Recruited patients were followed up for a duration of 12 weeks.

This study was approved by South Central—Hampshire A NHS Research Ethics Committee (part of the European Network of Research Ethics Committees).

### Data sources

Data was derived through secondary use of medical records; relevant data was extracted by HCPs and reported onto study specific questionnaires.

#### Baseline data

The baseline questionnaire collected information on demographic characteristics, anticoagulant regimen (total daily dose at treatment initiation), indication for treatment and prior anticoagulation/antiplatelet treatment. In addition, data on risk of stroke based on CHA_2_DS_2_-VASc (CHF/Left Ventricular Dysfunction, Hypertension, Age ≥ 75, Diabetes Mellitus, Stroke/TIA/ Thromboembolism History, Vascular disease history, Aged 65–74, Sex category) and bleeding based on HAS-BLED (Hypertension, Abnormal liver/renal function, Stroke history, Bleeding predisposition, Labile International Normalized Ratios (INRs), Elderly, Drug/alcohol usage) was collected. The HAS-BLED score was abridged for this study as labile INR is only relevant for warfarin patients.

#### Study outcomes

The primary objective was to estimate the incidence of major bleeding events (defined using International Society on Thrombosis and Haemostasis (ISTH) criteria [8] within gastrointestinal, urogenital and intracranial sites. Secondary outcomes included estimating all major bleeding (including within other sites) and clinically relevant non-major (CRNM) bleeds [9]. Reported bleeding outcomes were classified by an internal medical research fellow; any bleeding events which were slightly ambiguous were adjudicated by a second medical research fellow. All major bleeding events were further confirmed by an external independent medical expert reviewer.

### Sample size

Based on clinical trial 12 week cumulative incidence estimate of 0.7% for the primary outcomes of major bleeding (within gastrointestinal, urogenital and intracranial sites), a minimum sample size of 561 patients was calculated to provide sufficient precision (0.69%) to estimate cumulative incidence for these primary outcomes of interest for patients with NVAF taking rivaroxaban for the prevention of stroke and systemic embolism [4, 6].

### Statistical analysis

The analysable cohort for this paper were those patients with an indication of NVAF only. Incident reports were calculated on treatment (+5 drug half-lives [3 days] after stopping to account for drug elimination) during the 12 week observational period. Patients were censored according to the first of the following dates: end of 12 week observation period, loss to follow-up, death, first report of stopping treatment (+5 drug half-lives) or first report of outcome of interest. No imputation for missing values was conducted. Patients with missing data were excluded from the analysis for that specific variable. Statistical analyses of the baseline data were descriptive, exploratory, and generally limited to frequency tables or summary statistics (e.g. median + quartiles). The primary and secondary outcome measures are presented as unadjusted cumulative incidence (risk) and cumulative incidence rates, with corresponding 95% confidence intervals calculated using Binomial exact and Poisson exact, respectively. Data were analysed using STATA 15.0 software (StataCorp LLC, College Station, TX, USA).

## Results

**Fig 2:**
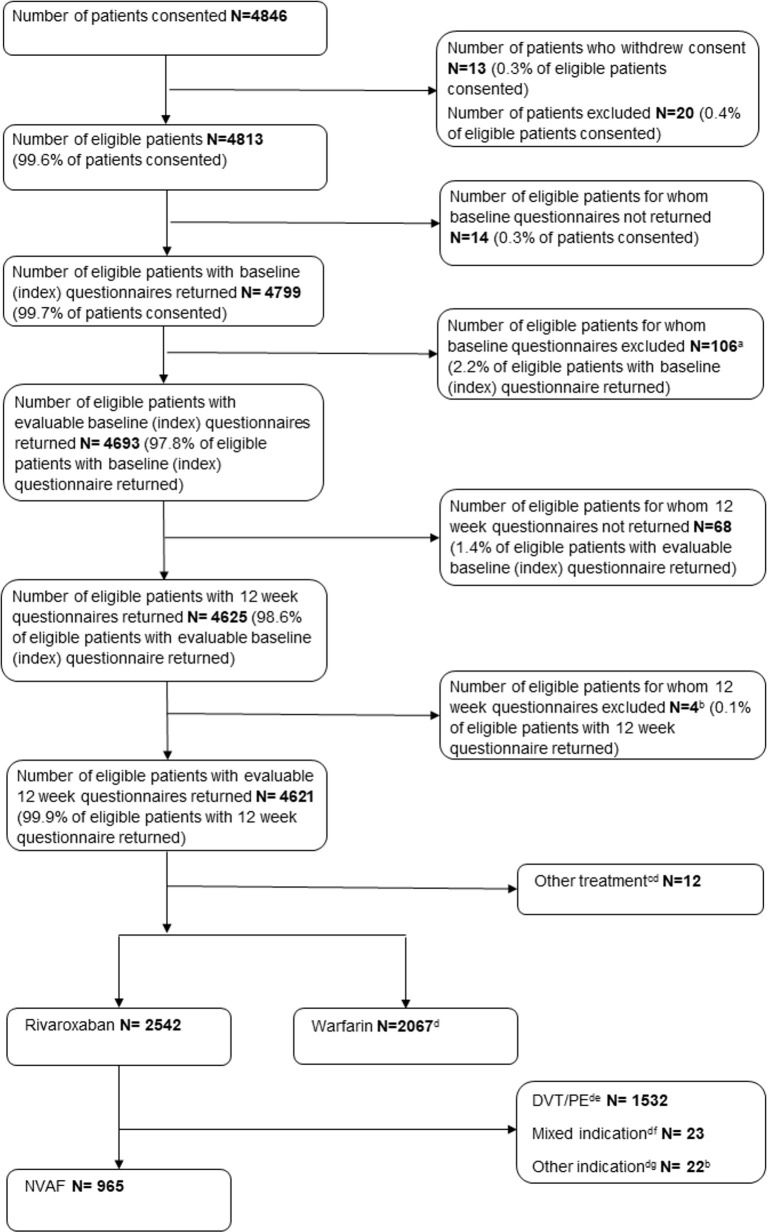
STROBE flowchart of the number of patients recruited over the course of the study. ^a^ Patient incorrectly identified (n = 87); did not start treatment (n = 13); decision to treat made in primary care (n = 4); questionnaire incomplete (n = 1); enrolled in another study (n = 1), ^b^ Patient incorrectly identified (n = 4), ^c^ Dalteparin (n = 10); enoxaparin (n = 2), ^d^ Not included in the analysable cohort, ^e^ Includes Treatment DVT (N = 860); Treatment of DVT+PE (N = 505); Prevent recurrent DVT/PE (N = 83); Other DVT/PE indication (N = 121), ^f^ Patients reported to have been treated for both AF and DVT/PE, ^g^ Patients for whom indication was ill-defined and/or off-label (Intracardiac thrombus (n = 3),Thrombophlebitis (n = 3), Thrombophlebitis superficial (n = 3), Atrial flutter (n = 2), Antiphospholipid antibodies (n = 1), Carotid artery thrombosis (n = 1), Cerebellar infarction (n = 1), Cerebrovascular accident (n = 1), Embolic stroke (n = 1), Left ventricular dysfunction (n = 1), Portal vein thrombosis (n = 1), Subclavian vein thrombosis (n = 1), Superior sagittal sinus thrombosis (n = 1), Thrombosis prophylaxis (n = 1), Not specified (n = 1)), DVT/PE = deep vein thrombosis/pulmonary embolism, NVAF = non-valvular atrial fibrillation.

A total of 4846 patients from 83 investigative sites (NHS Trusts) provided consent to participate in the study period from September 2013 to January 2016. Baseline and 12 week questionnaires were returned for 4625 (95.4%) patients; of these four (0.1%) were ineligible and excluded, resulting in 4621 evaluable patients. Rivaroxaban was prescribed for 2542 (55.0%) of these evaluable patients and 2067 (44.7%) evaluable patients were prescribed warfarin. In the rivaroxaban cohort, 965 patients were treated for NVAF, and most frequently initiated with a total daily dose of 20mg (Table 1). The remaining indications for prescribing rivaroxaban are provided in Fig 2.

**Table 1:** Baseline characteristics and posology of rivaroxaban.

Baseline characteristic	NVAF (N = 965)
Age (years), median (IQR)	76 (69, 83)
Gender (male), n (%)	517 (53.6)
BMI, median (IQR)	27.1 (23.9–31.1)^a^
*HAS-BLED*^b^, *n (%)*	
Hypertension	372 (38.6)
Abnormal renal function	15 (1.6)
Abnormal liver function	10 (1.0)
History stroke	298 (30.9)
History of bleeding or predisposition	162 (16.8)
Age ≥65 years	808 (83.8)
Drug therapy^c^	498 (51.7)
Alcohol (≥8 drinks/week)	37 (3.8)
HAS-BLED score, median (IQR)	2 (1–3)
Score, n (%)	
0	48 (5.0)
1	285 (29.5)
2	218 (22.6)
3	224 (23.2)
4	145 (15.0)
5	38 (3.9)
6	5 (0.5)
7	1 (0.1)
8	0 (0.0)
*CHA*_*2*_*DS*_*2*_*-VASc*, *n (%)*	
History congestive heart failure/left ventricular dysfunction	141 (14.6)
History hypertension	706 (73.2)
Age >75	559 (58.0)
Age 65–74	249 (25.8)
History stroke, TIA or thromboembolism	452 (46.9)
Vascular disease	259 (26.9)
Diabetes mellitus	181 (18.8)
Female sex	448 (46.5)
CHA_2_DS_2_-VASc score, median (IQR)	4 (3–6)
Score, n (%)	
0	8 (0.8)
1	69 (7.2)
2	107 (11.1)
3	168 (17.4)
4	195 (20.2)
5	171 (17.7)
6	164 (17.0)
7	58 (6.0)
8	22 (2.3)
9	2 (0.2)
Prior use of antithrombotic^d^ (*within 28 days of start of treatment*), n (%)	
Any	642 (66.5)
Aspirin	396 (41.0)
Warfarin	156 (16.2)
Dabigatran	5 (0.5)
Apixaban	1 (0.1)
Direct switching from prior antithrombotic^d^, n (%)	555 (57.5)
Aspirin	289 (52.1)
Warfarin	137 (24.7)
Dabigatran	5 (0.9)
Apixaban	1 (0.2)
**Starting total daily dose (mg), n (%)**	
<2.5	1 (0.1)
≥2.5, <5	1 (0.1)
≥5, <10	0 (0.0)
≥10, <20	176 (18.6)
≥20, <30	750 (79.5)
≥30	16 (1.7)
Missing	21 (-)
Median (IQR)	20 (20, 20)

^a^ BMI was missing for 221 patients (22.9%).

^b^ The HAS-BLED score was abridged for this study as labile INR is only relevant for warfarin patients.

^c^ Concomitant antiplatelets or non-steroidal anti-inflammatory drugs (NSAIDs).

^d^ Includes oral/parenteral anticoagulants, antiplatelets.

% where specified provided unless otherwise indicated.

### Baseline characteristics

The baseline demographic and clinical characteristics of the 965 rivaroxaban patients treated for NVAF are summarised in Table 1. The median patient age was 76 years; 58.0% were aged > 75 years and 53.6% were male. The median HAS-BLED score was 2 and the median CHA_2_DS_2_-VASc score was 4. A total of 555 (57.5%) patients were reported to have switched directly from another antithrombotic agent; over half of those switching directly to rivaroxaban switched from aspirin (n = 289; 52.1%) and approximately a quarter switched directly from warfarin (n = 137; 24.7%). Less than 1% switched from other DOACs.

### Outcomes

The number of major bleeds within each of the three primary sites of gastrointestinal, urogenital and intracranial were small (n = 2). The corresponding risk of major bleeding for each site within the 12 week observation period was 0.2%; 95% CI [0.0, 0.8] (Table 2). As major bleeding event counts were small (<10) within each of these three primary sites, incidence rates were not calculated.

**Table 2:** Incidence risk and rates of major of CRNM bleeding.

Bleeding Outcome*		N = 965		
		Number of patients	Risk (%) (95% CI)	Rate (per 100 patient years) (95% CI)
Major	Gastrointestinal	2	0.2 (0.0,0.8)	N/A^a^
	Urogenital	2	0.2 (0.0,0.8)	N/A^a^
	Intracranial	2	0.2 (0.0,0.8)	N/A^a^
	Critical organ site^b^	1	0.1 (0.0,0.6)	N/A^a^
	All^c^	10	1.0 (0.5,1.9)	5.5 (2.6,10.1)
CRNM^d^		41	4.3 (3.1,5.8)	22.7 (16.3,30.8)
Major bleed (All) and CRNM^e^		51	5.3 (4.0,7.0)	28.2 (21.0,37.1)

*Patients may have experienced more than one type of bleeding (e.g. major and clinically relevant non-major) within different sites, and so these counts are not mutually exclusive. In cases where multiple bleeding episodes have been reported within the same site, the most serious episode of bleeding was classified, and this bleeding classification with its associated event date was included in the analyses. Where events were reported but with no supporting event date, these patients were excluded.

^a^ rates were not calculated where event count n<10.

^b^ excluding all intracranial; bleeding events were considered to be critical if they occurred in intraspinal, intraocular, pericardial, intraarticular, intramuscular (with compartment syndrome), or retroperitoneal sites.

^c^ at least one major haemorrhagic event (irrespective of site).

^d^ at least one CRNM bleed.

^e^ at least one major haemorrhagic event (irrespective of site) and/or CRNM bleed.

The risk of major bleeding within other critical organ sites including intraspinal, intraocular, pericardial, intraarticular, intramuscular or retroperitoneal was 0.1%; 95% CI [0.0, 0.6]. For the composite outcome of all major bleeding (i.e. at least one major bleeding event, irrespective of site) the risk was 1.0%; 95% CI [0.5, 1.9]; the corresponding incidence rate was 5.5 events per 100 patients years; 95% CI [2.6, 10.1].

Clinically relevant non-major (CRNM) bleeding (irrespective of site) was more frequently reported than major bleeding in patients taking rivaroxaban for NVAF. The risk of CRNM bleeding was 4.3%; 95% CI [3.1, 5.8] corresponding to an incidence rate of 22.7 per 100 patient years; 95% CI [16.3, 30.8]. For the composite outcome of all major and/or CRNM bleeds the risk was 5.3%; 95% CI [4.0, 7.0] and the rate was 28.2 events per 100 patient years; 95% CI [21.0, 37.1].

## Discussion

The primary objective of the study was to estimate the incidence (separately) of major bleeding within gastrointestinal, urogenital and intracranial sites during the 12 week observation period. Within this analysis, we examined patients with an indication of NVAF only. The risk in each of the three sites was low; 0.2%; 95% CI [0.0, 0.8]. Findings for gastrointestinal and intracranial bleeding from this study are lower than that observed in the ROCKET-AF trial (3.2% and 0.8%, respectively) and also the large prospective XANTUS study (0.8% and 0.4% respectively) although they were longer studies (median observation period 707 and 366 days respectively) [4, 10]. The only urogenital site major bleed reported within the ROCKET-AF study was for macroscopic haematuria, with a cumulative incidence of 0.4% [4]. Subsequent post-hoc analyses of major bleeding events occurring in ROCKET-AF found that ‘increased/prolonged menstrual or abnormal vaginal bleeding’ occurred in three of the 135 women experiencing a major bleed on rivaroxaban (2.2%) [11].

Secondary outcomes included estimates of major bleeding within other sites and CRNM bleeding. The risk of all major bleeding (at least one major haemorrhagic event, irrespective of site) was 1.0%; 95% CI [0.5, 1.9]; the corresponding rate was 5.5 events per 100 patient years; 95% CI [2.6, 10.1]. The rate of major bleeding in this study was higher than that observed in both the ROCKET AF trial and the XANTUS study; 3.6 events per 100 patient years and 2.1 events per 100 patient years respectively [4, 10]. A meta-analysis of real-world observational studies of rivaroxaban in NVAF patients concluded that pooled rates of major bleeding with rivaroxaban were generally low (3.32 per 100 patient years; 95% CI [2.28, 4.25]) and consistent with those reported in its pivotal randomized controlled trial [12].

The risk of CRNM bleeding in the ROSE study was 4.3%; 95% CI [3.1, 5.8] corresponding to an incidence rate of 22.7 events per 100 patient years; 95% CI [16.3, 30.8]. This is higher than the rate for the ROCKET AF (11.8) and XANTUS (15.4) studies [4, 10]. For the composite outcome of all major and CRNM bleeds the risk was 5.3%; 95% CI [4.0, 7.0] and rate was 28.2 events per 100 patient years; 95% CI [21.0, 37.1]. This is higher than the rate of major and non major clinically relevant bleeding in ROCKET AF; 14.9 events per 100 patient years [4].

Although the overall rates of major and CRNM bleeding within the ROSE study appear higher than in both clinical trials and other published observational studies, patients enrolled in our study had a moderate baseline risk (~2/100 patient-years) for major bleeding (median HAS-BLED score 2). Direct comparison of bleeding risk is not possible since HAS-BLED scores were not calculated for the ROCKET AF and XANTUS studies. However, HAS-BLED was calculated in a subset of the XANTUS study, where the baseline bleeding risk (median HAS-BLED score 2 [IQR 1–2]) was consistent with the ROSE findings [13]. We acknowledge that the low numbers of bleeding events in the ROSE study and differences in study design compared to these studies, in particular with respect to review of medical records in ROSE, could contribute towards discrepancies in the incidence of bleeding outcomes. Therefore, direct comparisons should be interpreted with caution.

As with other post authorisation safety studies which rely on secondary data collection, a limitation of this study design is the potential for underreporting or selective reporting of particular events and missing data. However, all relevant cases of haemorrhage were adjudicated and if necessary additional information was requested from the specialist HCPs by use of follow up questionnaires. In SCEM, exposure is based on prescription data as recorded in medical records, however, as with many observational studies, the degree of patient compliance in taking the prescribed medication cannot be fully ascertained. The aim of the study design was to obtain a representative sample of patients across England and Wales. For both participating and non-participating sites, data was collected and compared in order to explore representativeness. Whilst there appeared to be no obvious differences for some of these indicators, such as geographical distribution, type of trust, or proportions of trusts with at least one hospital with teaching status, as was expected, indicator data for adoption of new medicines based on hospital density, population density and rivaroxaban sales were higher for participating compared to non-participating sites. Furthermore, it is known that medicines management policies determine which trusts will prescribe new treatments; in this study, a slightly higher proportion of participating trusts had guidelines for use of rivaroxaban available compared to non-participating sites.

In addition to the very high response rate achieved in the ROSE study (96% of eligible patients consented had an evaluable 12 week questionnaire returned [Fig 2]) a key strength of this study methodology is the ability to identify patients treated in a secondary care setting; this allows data to be collected on a more diverse patient population, including patients of greater medical complexity, and patient populations that are often excluded from clinical trials. For those patients whose treatment was initiated in secondary care, information on the short term risk of bleeding was collected from the very beginning of treatment, thus including the early risk window and filling an evidence gap not addressed by other studies. The methodology also allows the identification of the inception cohort in a timely fashion and facilitates the collection of highly detailed information. In those cases where additional information is required, further requests for information can be made to the relevant prescriber, for case ascertainment. This degree of detail enabled the calculation of relevant risk scores, and the application of outcome definitions used in clinical trials, such as the ISTH bleeding classification.

In conclusion, in terms of the primary outcome risk of major bleeding within gastrointestinal, urogenital and intracranial sites during the 12 week observation period, the risk estimates in this UK secondary care NVAF rivaroxaban user population were low (<1%), and consistent with risk estimated from clinical trial data and routine clinical practice. The SCEM design has been shown to provide a suitable framework to evaluate the safety of newly marketed medicines in the secondary care setting.
